# Notes and News

**Published:** 1889-05-04

**Authors:** 


### Notes and News.

COLLECTED AND COMMUNICATED.

The meeting last Saturday to consider the penny-a-week hospital scheme, at Holloway Hall, was a very crowded affair. The Lord Mayor presided, and made an excellent speech. Sir A. K. Rollit also spoke. What was chiefly noticeable, though, was the crowd which congregated inside and outside the hall. It showed what a great interest the people now take in the Hospital Saturday movement.

Amongst the pictures of interest which our readers must notice in the forthcoming Academy, is a portrait of Mr. Jaffray, by John Pettie, R.A. Mr. Jaffray is the founder of the hospital which bears his name at Birmingham, and also proprietor of the *Birmingham Post*. The portrait is an excellent likeness and a notable work of art. It ought to find a place in the hall of the Jaffray Hospital.

In reply to many enquiries, we have pleasure in stating that Mr. Burdett's biography of the Prince and Princess of Wales, entitled "Prince, Princess, and People," is published by Messrs. Longmans, Green, and Co., and may be had at all libraries throughout the country. As the book is in great demand, those who desire to obtain an early copy from a library had better write for it at once. The book contains much that is especially interesting to members of the medical profession, nurses, and others who take an active interest in the social well-being and happiness of the people.

There is to be a commission to enquire into the working of the present Act which makes vaccination compulsory; and the enemies of vaccination are more or less triumphant. What the enquiry will prove, of course, remains to be seen, but, at least, its proceedings will be of great interest, and its conclusions will be valuable. It was time some such step was taken, for the difference of opinion even amongst medical men on this subject was great, and the law was not enforced in many places. A law which is allowed to become void breeds disdain for law in the abstract; it is much better that a rule which is not upheld should be publicly repealed. We can now hope that the present state of indecision will shortly vanish, and that magistrates and medical men will be able to act in future with decision when the question of compulsory vaccination is brought before them.

St. Mary's Hospital festival dinner is to be presided over this year by Mr. John Aird, M.P. for North Paddington. As the charity is situated in North Paddington, it is most fitting that the member for that division should take the chair. It is hoped that the hon. gentleman's wealthy neighbours in South Paddington will rally round him on this occasion in the interest of a charity which does so much for their poorer neighbours in the northern division of the borough. The dinner is to take place at the Hotel Métropole on May 25, the objects for which it is to be held being to increase the annual subscription list, which is so small as to prejudice the financial security of the institution, and to augment the fund which was inaugurated at the special festival dinner last year, when Lord Randolph Churchill took the chair, for the extension of the hospital into Praed-street. It would be well if the Parliamentary representatives of other metropolitan boroughs were to emulate the active interest taken by the members for Paddington in the hospital in their constituency. Paddington has every reason to be proud of the help its members have given to St. Mary's Hospital.

Lord Hartington's visit to Sutherland to lay the memorial-stone of the infirmary was, unfortunately, marred by an accident. Just at the commencement of the ceremony the platform collapsed, and several people were slightly injured. Fortunately they have now all recovered. Lord Hartington's speech was very short, and the proceedings were altogether upset by the accident. The Hartley Memorial Wing is to cost £11,000, of which £9,000 is already subscribed.

The *Glasgow Evening News* has been giving a long account of the growth and cost of the Belvidere Hospital for infectious diseases. It is all very well to refer to the huge sums which such buildings cost, but it is no use whatever to take half measures in dealing with fever cases. Apart from the £26,000 spent on the building and site of the fever hospital in Parliamentary-road, Belvidere has cost the ratepayers £120,235. According to the accounts for the year ended May, 1888, the cost of management was £17,768 17s. 10d. The total number of patients treated for the same period was 2,760. Slightly over six weeks was the average term of residence, and the cost per head £6 6s. 1d. The estimated expenditure for the current year is over £20,000, including £4,510 in salaries and wages to staff, and £5,450 for food of patients and officials.

The twenty-eighth annual report of the Glasgow Hospital for Skin Diseases states that during the past year the number of new cases admitted has been 1,492, as against 1,159 in 1888—the total number treated since the opening of the institution in 1861 being 33,948. The cases admitted into the cutaneous wards of the Western Infirmary during the year have been 120, as against 93 in 1888; and the whole number received since the opening of these wards, 14 years ago, has been 1,255. The number of students who availed themselves of the instruction provided at the hospital has been greater than on any previous year, being 91, as against 55 in 1888. This is exclusive of medical men, who have availed themselves to a much greater extent than formerly of the privilege of seeing the practice of the hospital, thus showing the increasing interest which is being taken in this important class of affections. There is a deficiency in the treasurer's report of £578.

The Local Government Board has issued a special report of diarrhœa and diphtheria, by Dr. Ballard and Dr. G. B. Longstaff. Dr. Ballard has supplied a basis of fact, in place of much that was speculative in our conceptions of so-called "summer diarrhœa," and from a store of statistical, clinical, and pathological data that he has formulated, a truer and more definite judgment respecting the disease, its nature and causes, than had before been attained. As to its nature he indicates that it is no mere local malady of the intestines, showing itself by flux or inflammation, but that it is rather a general constitutional affection proper, to be classed among the "acute specific diseases." Dr. Longstaff's essay sets forth, as the result of abundant and careful examination of mortality statistics, the facts as to the distribution of fatal diphtheria in the districts of England and Wales during the 26 years ending with 1880; and he shows, in the first place, very notable varieties of incidence of the disease upon the several counties not applicable by any geographical or geological consideration. He brings into strong relief evidence to show that the disease has always displayed a marked tendency to prevail in sparsely populated districts rather than in the centres of population, and that as years have gone on this tendency has become less and less marked, so that in later years the chief urban districts seem to be approaching nearer than before the rural districts in their rate of suffering by this disease. The report will be of great interest to all medical men.

The following vacancies are announced this week, the amount of the salary in each case being given after the name of the institution:

**Resident Medical Officers.**

Birmingham General Dispensary. Surgeon. — £150, rooms, &c.
Rotherham Hospital. House Surgeon.—£100, board, &c.
Willesden Local Board. Medical Officer.—£100.
Public Dispensary, 59, Stanhope street. Medical Officer. £105, rooms, &c.
Morningside Asylum. Junior Assistant Physician.
Swansea Lodge of Oddfellows. Medical Officer.
Warwick Lunatic Asylum. Medical Superintendent.—£600, house, &c.
Ipswich Hospital. Assistant House Surgeon.—£20, board, &c.

**Non-Resident Medical Officer.**

Victoria University, Leeds. Lecturer on Diseases of Women and Children.

---

An inquest was held at Liverpool last week on the body of Elizabeth Jones, aged thirty-five. The deceased's brother some time ago brought a cat from Cavan, where hydrophobia was prevalent. In February last the cat became ill, and the deceased, who attended to it, was bitten in the thumb. She afterwards complained of pain, and about a week ago she developed symptoms of hydrophobia, and died after two days' terrible suffering. A verdict was returned in accordance with the medical testimony.

---

The annual meeting of the supporters of Carmarthenshire Infirmary passed off well. The expenditure last year was £989, leaving a balance in the treasurer's hands of over £100. The house surgeon's report was read, from which it appeared that the number of patients treated during the past year was 975, of which 147 were in-patients and 826 out-patients, this being an increase of 316 over last year. There had been five deaths of in-patients. Since the infirmary was opened 37,805 patients had been treated. The only change in the staff last year was the appointment of Miss MacIntyre as matron in place of Miss Lambert, removed to Southport.

---

The Charity Organisation Society has issued a memorandum on the hospital question, and tells us once more that hospitals are abused. It is a well-known fact, and one against which the authorities have struggled long, and for which the charitable public is chiefly to blame. The figures quoted in the memorandum are likely to astonish the uniniated, to whom over a million of hospital patients for the metropolis seems a liberal allowance. The charity organisers do not tell us what they are going to do to try and reduce this number by cutting out the unworthy and leaving in the worthy. We suppose they are not going to stop short at a memorandum, and we wish them practical success in dealing with this question.

---

The Duke of Rutland presided at the annual meeting of Leicester Infirmary. The number of in-patients treated during the past year had been smaller than in 1887, the daily average in the house had been considerably larger, and their stay longer in 1888 than in the previous year. This was to be accounted for mainly by the increased number of fever patients. No fewer than 183 cases had been treated during the year; by far the largest number since 1857. It was well known that these cases were very tedious and expensive, and it was for the most part owing to the ncrease in this department that the ordinary expense of last year exceeded the income by £1,000, a deficiency which the board had, with reluctance, met by the realisation of a portion of the invested capital. The Duke of Rutland said he hoped the Children's Hospital would be well endowed before being opened.

Every now and then the lay papers get hold of some feat of surgery and laud it to the skies. A little while ago it was a case of transfusion, as though there was only one case of transfusion in a dozen years! Now it is a case of nerve-grafting, which is reported to the *Manchester Guardian.* A provincial surgeon, in removing a tumour from a patient's arm, removed some of the nerve, and there was consequent loss of sensation. The surgeon obtained a piece of healthy nerve from a lately amputated leg, and replaced the lost portion in the arm, and after thirty-six hours sensation returned. It is a pretty operation this, and one that appeals to the sense of the marvellous in the general public. Many years ago Hardy, of Liverpool, made a girl a new nose out of the top joint of one of her fingers, and this also was loudly applauded by the newspapers; but, as a rule, the real triumphs of surgery are unheeded by the press.

---

The report of the annual meeting of the governors of Nottingham General Hospital is satisfactory in most particulars. The total receipts last year amounted to £10,539; the principal items of expenditure were: Matron's department, for meat £1,382 11s. 1d.; for milk, etc., £616 12s. 6d.; for butter, cheese, and eggs £411 7s. 8d.; for ale, stout, tea, coffee, and cocoa £286 17s. 1d.; for coal, gas, and water £628 2s. 3d. (£4,032 4s. 2d.); wines and spirits £84 0s. 2d.; apothecary's department, £782 11s. 6d.; salaries, wages, and commission, £1,633 9s. 2d.; miscellaneous expenses, for printing, district rate, fabric and repairs, etc., £1,455 4s. 7d.; making £7,987 9s. 7d.; by purchase of property, £1,102 18s. 2d. The in-patients numbered 1,419 and the out-patients 7,732 for the year. There is a continued decrease in the number of annual subscribers, and it will be generally admitted that this falling off in what must always be the main source of revenue for charitable institutions is not creditable to Nottingham. The annual subscriptions form the only income the governors can certainly rely upon. Legacies may come, if benevolent people happen to die in the year, but legacies cannot be depended upon. The receipts from both the Hospital Sunday and Hospital Saturday collections keep up well, but the receipts from these two sources only amount to about a couple of thousand pounds, while the expenses are, roughly speaking, about eight thousand.

---

The forthcoming "Al Fresco Fayre" at Albert Hall, in aid of the Grosvenor Hospital for Women and Children, has roused interest in the work done by that institution. As at present carried on, the work is divided into two departments, an out-patient department for women and children, where from between fifty and eighty are seen each day, and an in-patient department, for the treatment only of diseases peculiar to women. For the latter there are four small wards, each with three beds, and two private wards, each having one bed for special cases. The advantages which such a hospital offers are manifest. There are innumerable women not sufficiently well off to secure the necessary medical aid, efficient nursing, suitable food, and quiet in their own homes, who can yet well afford to pay a small sum weekly for such advantages, and prefer to do so, rather than go into the large public hospitals as gratuitous patients. Captain Howard is head manager of the Fayre, and will avoid the blemishes which marred the Ice Carnival. Among the stallholders will be the Viscountess Coke, the Marchioness of Hertford, Mrs. Bancroft, the Lady Constance Howard, the Viscountess Torrington, Lady Coleridge, Lady Gough, Mrs. Ronalds, the Duchess of Marlborough, Lady Margaret Seymour, Mrs. Arthur Weguellin, the Countess F. Lutzow, Lady Jane Taylor, Lady Decies, the Hon. Mrs. Evelyn Ellis, Hon. Mrs. Stopford, the Hon. Mrs. A. J. Ram, Mrs. W. Cunard, and Lady Dalrymple Elphinstone.

